# Association of Interleukin-1 Receptor Antagonist ( *IL-1RA* ) Gene Polymorphism with Community-Acquired Pneumonia in North Indian Children: A Case–Control Study

**DOI:** 10.1055/s-0043-1770056

**Published:** 2023-06-16

**Authors:** Neha Verma, Shally Awasthi, Anuj K. Pandey, Prashant Gupta

**Affiliations:** 1 Department of Pediatrics, King George's Medical University, Lucknow, Uttar Pradesh, India; 2 Department of Microbiology, King George's Medical University, Lucknow, Uttar Pradesh, India

**Keywords:** community-acquired pneumonia, interleukin-1 receptor antagonist, variable number of tandem repeats, polymerase chain reaction, odds ratio, adjusted odds ratio

## Abstract

**Background** Community-acquired pneumonia (CAP) is the leading cause of death in children < 5 years of age. The primary objective of the study was to assess the association of *IL-1RA* gene polymorphism in children aged 2 to 59 months with CAP and the secondary objective was to assess the association of gene polymorphism with mortality among hospitalized CAP cases.

**Study Design** This case–control study was conducted in a tertiary teaching institute in Northern India. Hospitalized children aged 2 to 59 months with World Health Organization-defined CAP were included as cases after parental consent. Age-matched healthy controls were recruited from the immunization clinic of the hospital. Genotyping was done using polymerase chain reaction to analyze the variable number of tandem repeats of *IL-1RA* gene polymorphism.

**Result** From October 2019 to October 2021, 330 cases (123, 37.27% female), and 330 controls (151, 45.75% female) were recruited. Genotype A2/A2 of the *IL-1RA* gene was found to be associated with the increased risk for CAP children with adjusted odds ratio (AOR) of 12.24 (95% confidence interval [CI] 5.21–28.7, *p* < 0.001). A2 and A4 alleles were also found to be at risk for CAP. A1/A2 genotype was found to be protective for CAP with an AOR of 0.29 (95% CI 0.19–19.0.45). The genotype A2/A2 and A2 allele of *IL-1RA* gene was associated with child mortality with CAP cases.

**Conclusion** In *IL1RA* gene, A2/A2 genotype and A2 allele were associated with increased risk of CAP and A1/A2 were found to be protective for CAP. The genotype A2/A2 and A2 was associated with CAP mortality.

## Introduction

Community-acquired pneumonia (CAP) is one of the most common infectious diseases of the lower respiratory tract in children less than 5 years of age. Based on burden of CAP, India shares 23% of global cases. 1 In the year 2021, it was reported that 14% of all death of children under the age of 5 years, worldwide, was due to CAP. 1 CAP is an infective inflammation of lung parenchyma caused by bacterial or viral pathogens both and less commonly by fungi. 1 Various researches have suggested that the genetic variables can induce exaggerated and persistent inflammatory responses in certain cases which can increase the severity of CAP. 2 3 4 Mortality is mostly due to complications associated with severe CAP. 5 6 Mechanisms underlying and influencing progression from pneumonia to severe pneumonia or complications are not clearly understood. Studies have reported that comorbid conditions like malnutrition, anemia, and hypoxemia increase the risk of CAP-associated child mortality. 7 8

One of the proinflammatory cytokines activated in response to CAP is interleukin (IL)-1. IL-1 has a natural antagonist, IL-1 receptor antagonist (IL-1RA). Both IL-1 and IL-1RA binds to the same receptor called IL-1R, thereby modulating the proinflammatory activity of IL-1. 3 9 *IL-1RA* gene is responsible for production of IL-1RA and is mapped on long arm of 2nd chromosome in human (2q14.1). The polymorphic region within intron 2 of the *IL-1RN* gene contains a variable number of tandem repeats (VNTRs) of 86 base pair (bp). 2 3 Penta allelic polymorphic site have been reported of *IL-1RA* gene containing 2 to 6 repeats. It is possibly that the number of repeats may influence gene transcription and protein synthesis. The most frequently studied alleles are A1 and A2. There is evidence that allele 2 of the *IL-1RA* gene is associated with increased susceptibility or more severe outcome in chronic inflammatory diseases such ulcerative colitis, systemic lupus erythematosus, and alopecia areata. 10 11 12 13

Therefore, the current study was undertaken with the primary objective to assess the association of *IL-1RA* gene polymorphism in children aged 2 to 59 months with CAP and the secondary objective was to assess the association of gene polymorphism with mortality among hospitalized cases.

## Material and Methods

### Study Setting

This study was conducted at the Department of Pediatrics, King George's Medical University, Lucknow, Uttar Pradesh, India, from 2019 to 2021 after obtaining ethical clearance from the Institutional Ethics Committee. Written informed consent was obtained from the parents before the recruitment of both cases and controls.

### Study Design

This was a case–control study. Hospitalized cases were recruited from the pediatrics wards. The World Health Organization (WHO) definition was used to define cases. As per the WHO classification, children with presence of cough and/or difficulty breathing, where the respiratory rate is above age-specific range, with or without chest indrawing are classified as pneumonia. 14 However, children with presence of general danger signs, namely, not able to drink, persistent vomiting, convulsions, lethargic or unconscious, stridor in calm child, or severe malnutrition, are classified as severe pneumonia or very severe disease. Healthy controls were recruited from immunization clinic of pediatric department.

### Inclusion/Exclusion Criteria of Cases

All hospitalized children aged 2 to 59 months were screened. Children diagnosed with WHO-defined CAP who had radiological abnormalities of CAP were included as cases in this study. 15 Children with radiological CAP along with pleural effusion/empyema/pneumothorax needing drainage or those with acute respiratory distress syndrome (ARDS) or septic shock, were categorized as complicated CAP. 16 Those children who had none of the above-listed complications or had minimal synpneumonic effusion not needing drainage were categorized as uncomplicated CAP.

The exclusion criteria of the cases were as follows: (1) prior enrollment as a case in present study, (2) symptomatic for > 14 days, (3) history of known asthmatic or chronic lung diseases, (4) known immunodeficiency patient, and (5) patient receiving treatment of tuberculosis.

### Inclusion/Exclusion Criteria of Controls

Age- (±2 months) and gender-matched healthy children were recruited from the immunization clinic within 1 week of the recruitment of cases. Excluded were those children who had previous history of pneumonia or any other acute or chronic respiratory diseases.

### Demographic and Socioeconomic Data

All data were collected on a predesigned, pretested questionnaire. Parents/caregivers were interviewed for the collection of demographic details like age, gender, residence, and educational qualification. All the clinical details such as diagnosis, comorbid conditions, and presenting signs and symptoms were abstracted from the patients' medical records. The immunization record of recruited children were reviewed and noted. Revised Kuppuswamy's scale 17 was used to assess the socioeconomic status (SES). SES was assessed by education, occupation of the head, and household income of the family.

### Anthropometric Measurements

Anthropometric measurements were taken. Weight (in kg) was recorded by an electronic weighing machine. The unit was standardized by calibrating it to zero before each measurement. Stadiometer was used to measure the height (in cm) for children who were > 2 years, while an infantometer was used for younger children. All the digits were corrected to one decimal unit. Weight for age, height for age, and weight for height were calculated on the basis of anthropometric measurement. 18

### Sample Size

The sample size was calculated on the basis of a previous study in the similar study setting on children with CAP by using online OSSE (Online Sample Size Estimator) software. The prevalence of the A2 allele of *IL-1RA* gene for cases was reported to be 16.42% in children of same ethnicity in Lucknow. 3 The allele A2 was reported to be associated with an increased risk of adverse outcome (AO) in CAP children. However, for controls, the minor allele frequency of A2 allele was taken from another study of the same geographic region, which was 30.61%. 19 The power of the study was taken as 95% with 5% significant level. The calculated sample was 232 cases and 232 controls by using the 1:1 ratio for calculation.

### Sample Processing Molecular Work

Whole blood was collected from each participant under aseptic conditions. Two milliliter blood was drawn in ethylenediaminetetraacetic acid. Genomic deoxyribonucleic acid (DNA) was isolated by using standard phenol chloroform method. Purity of DNA was checked by spectrophotometer at 260/280 nm. Polymerase chain reaction (PCR) amplification was performed by reported primers. 20 The PCR conditions were set to 95°C in initial denaturation for 3 minutes, followed by 35-cycle annealing at 59°C for 1 minute, followed by extension at 72°C for 1 minute and final extension at 72°C for 10minutes. Band sizes of the amplified PCR products were as follows: 410 bp (four repeats) was allele A1, 240 bp (two repeats) allele A2, 500 bp (five repeats) allele A3, 325 bp (three repeats) allele A4, and 595 bp (six repeats) was allele A5.

### Statistical Analysis

All the data was double entered in MS Excel. Statistical analysis was done by using Statistical Package of Social Science (SPSS version 20). The mean and standard deviation (SD) for continuous data and percentage for categorical data were calculated. Chi-square test was used for categorical and Student's *t* -test was used for continuous variables. Also, Fisher's exact test was used when the cell frequency was less than 5. Crude odds ratio (OR) with 95% confidence interval was calculated for the outcome variable. Hardy–Weinberg equilibrium was used separately in both, cases and controls, for the allelic distribution. A *p* -value of < 0.05 was taken as statistically significant using two-tailed distribution. The strength of association between genotype and outcome was expressed by the ORs. Unconditional logistic regression model was used to assess the association of common genotype of *IL-1RA* gene with CAP controlling for socioeconomic factor, pneumococcal vaccine (PCV) and measles immunization, height for age, and SES which had significant association with CAP.

## Results

**Fig. 1:**
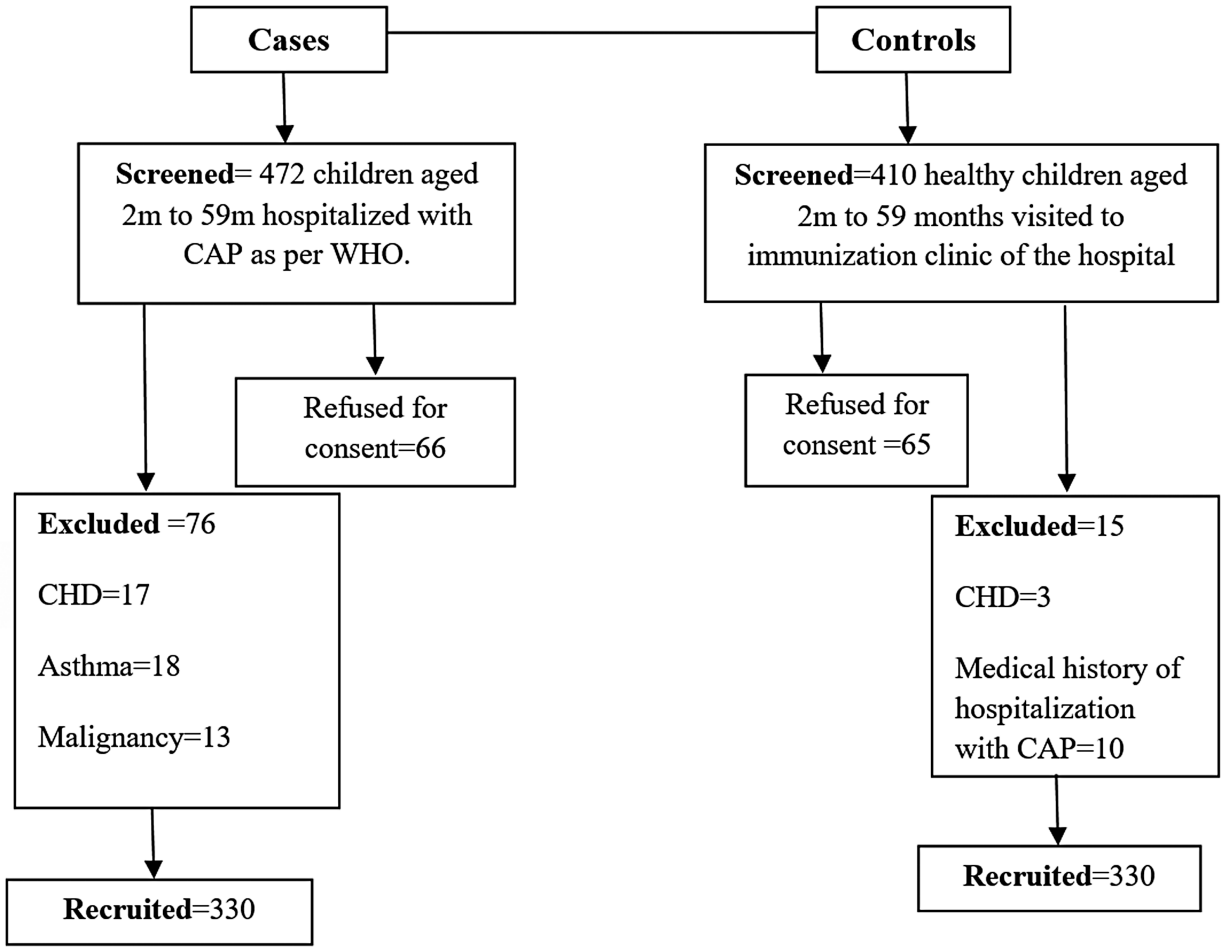
Flowchart of the study. CAP, community-acquired pneumonia; CHD, congenital heart disease; TBM, tubercular meningitis; WHO, World Health Organization.

Recruitment of the participants in the study was done from October 2019 to October 2021. In this duration, age- and gender-matched, 330 cases and 330 controls were recruited into the study. The flowchart of the study was shown in Fig. 1 . The mean age of the cases was 14.8 ± 13.5 months and control was 13.6 ± 12.8 months. The sociodemographic profile of study participants is given in Table 1 .

**Table 1:** Demographic characteristic of all included subjects

Variables, *n* (%)	Cases ( *N* = 330)	Control ( *N* = 330)	*p* -Value
Age (mo)			
2–11	169 (48.8)	177 (51.2)	0.4
12–23	84 (48.3)	90 (51.7)	
24–59	77 (55.0)	63 (45.0)	
Gender (female)	123 (37.57)	151 (45.75)	0.2
Weight (kg) (mean ± SD)	7.2 ± 3.2	8.01 ± 3.2	0.24
Height (cm) (mean ± SD)	66.3 ± 19.0	66.3 ± 17.5	0.23
Immunization against PCV	39 (11.8)	231 (70.0)	**< 0.001**
Immunization against DPT	237 (71.8)	261 (79.1)	0.03
Immunization against measles a	84/171 (49.1)	156/166 (94.0)	**< 0.001**
Residence (rural)	299 (90.6)	57 (17.3)	**< 0.001**
Separate cooking space	274 (83.0)	330 (100.0)	**< 0.001**
Firewood/kerosene	127 (37.6)	36 (10.9)	**< 0.001**
Gas	203 (61.5)	294 (89.1)	
Ever breast-fed	295 (89.4)	275 (83.3)	0.02
Duration of breastfeeding (mo) (mean ± SD)	7.17 ± 7.5	10 ± 12.72	0.006
Weight for age (≤ –3 SD)	109 (33%)	31 (9.4%)	**< 0.001**
Height for age (≤ –3 SD)	141 (42.7)	72 (21.8)	**< 0.001**
Weight for height (≤ –3 SD)	77 (23.3)	13 (3.9)	**< 0.001**
Socioeconomic status			
Lower	169 (51.2)	45 (13.6)	**< 0.001**
Middle/Upper	161 (48.8)	285 (86.4)	

Abbreviations: DPT, diphtheria, pertussis, and tetanus; PCV, pneumococcal vaccine; SD, standard deviation.

a Immunization against measles was calculated of children whose age was more than 9 months.

Genotypic and allelic distribution of *IL-1RA* gene polymorphism among cases and controls is given in Table 2 . The allelic frequencies of the *IL1RA* gene in cases and controls were as follows: A1 = 57.42%, A2 = 26.81%, A3 = 6.81%, and A4 = 8.93% or A1 = 66.06%, A2 = 17.5%, A3 = 10.6%, and A4 = 5.7%. The allele A5 was not found among cases and control. Based on the analysis of genotypic distribution of *IL-1RA* gene, A1/A1 genotype was most commonly found in both the cases and controls (46.06%, 152/330 and 40.30%, 133/330). Increased odds of CAP was found in children carrying A2/A2 genotype whereas A2 alleles of this variant was 1.7 times more susceptible to CAP. A4 allele of *IL-1RA* gene was also found to be associated with increased odds of CAP in children. Similarly, A2/A2 and A4/A4 genotype and A2 allele of *IL-1RA* gene were found to be associated with mortality in CAP children ( Table 3 ). The genotype A2/A2 and A2 alleles were found to be associated with complicated CAP ( Table 4 ).

**Table 2:** Genotype and allele distribution of *interleukin-1 receptor antagonist* gene among cases and controls

Serial no.	Genotypes, *n* (%)	Cases, *N* = 330	Controls, *N* = 330	Crude OR (95% CI)	*p* -Value
1	A1/A1	152 (46.06)	133 (40.30)	1.26 (0.92–1.72)	0.15
2	A1/A2	35 (10.60)	94 (28.48)	0.29 (0.19–0.45)	**< 0.001**
3	A1/A3	9 (2.72)	50 (15.15)	0.15 (0.07–0.32)	**< 0.001**
4	A1/A4	31 (9.39)	26 (7.87)	1.21 (0.7–2.09)	0.57
5	A2/A2	61 (18.48)	6 (1.81)	**12.24 (5.21–28.7)**	**< 0.001**
6	A2/A3	10 (3.03)	6 (1.81)	1.68 (0.60–4.69)	0.44
7	A2/A4	10 (3.03)	4 (1.21)	2.54 (0.79–8.20)	0.17
8	A3/A3	11(3.33)	4 (1.21)	2.81 (0.88–8.9)	0.11
9	A3/A4	4 (1.21)	6 (1.81)	0.66 (0.18–2.37)	0.75
10	A4/A4	7 (2.12)	1 (0.30)	7.13 (0.87–58.3)	0.07
	**Alleles,** ***n*** **(%)**	**Cases,** ***N*** ** = 660**	**Controls,** ***N*** ** = 660**	**Crude OR (95% CI)**	***p*** **-Value**
11	A1	379 (57.42)	436 (66.06)	0.69 (0.55–0.86)	**< 0.001**
12	A2	177 (26.81)	116 (17.5)	**1.71 (1.31–2.23)**	**< 0.001**
13	A3	45 (6.81)	70 (10.60)	0.61 (0.41–0.91)	0.01
14	A4	59 (8.93)	38 (5.7)	**1.60 (1.05–2.45)**	**0.03**

Abbreviations: CI, confidence interval; OR, odds ratio.

**Table 3:** Genotype and allele distribution of *IL1RA* gene among uncomplicated and complicated severe community acquired pneumonia

Serial no.	Genotypes, ( *N* = 330) (%)	Complicated, *n* = 209 (%)	Uncomplicated, *n* = 121 (%)	Crude OR (95% CI)	*p* -Value
1	A1/A1 ( *n* = 152)	62 (29.6)	90 (59.2)	0.14 (0.08–0.24)	< 0.001
2	A1/A2 ( *n* = 35)	28 (13.4)	7 (5.7)	2.5 (1.06–5.95)	**0.04**
3	A1/A3 ( *n* = 9)	2 (0.95)	7 (5.7)	0.15 (0.03–0.77)	**0.02**
4	A1/A4 ( *n* = 31)	22 (10.52)	9 (7.4)	1.46 (0.65–3.29)	0.46
5	A2/A2 ( *n* = 61)	58 (27.75)	3 (2.5)	15.10 (4.6–49.43)	**< 0.001**
6	A2/A3( *n* = 10)	10 (4.7)	0 (0)	NA	
7	A2/A4 ( *n* = 10)	9 (4.3)	1 (0.83)	5.4 (0.67–43.170	0.09
8	A3/A3 ( *n* = 11)	10 (4.8)	1 (0.83)	6.03 (0.76–47.10)	0.06
9	A3/A4 ( *n* = 4)	4 (1.91)	0 (0)	NA	
10	A4/A4 ( *n* = 7)	4 (1.91)	3 (2.5)	0.76 (0.16–3.48)	0.71
Alleles, *N* = 660 (%)					
11	A1 ( *n* = 379)	176 (42.10)	203 (83.9)	0.13 (0.09–0.20)	< 0.001
12	A2 ( *n* = 177)	163 (39.0)	14 (5.8)	10.4 (5.86–18.49)	**< 0.001**
13	A3 ( *n* = 45)	36 (8.6)	9 (3.7)	2.4 (1.15–5.15)	0.06
14	A4 ( *n* = 59)	43 (10.3)	16 (6.6)	1.6 (0.89–2.94)	**0.14**

Abbreviations: CI, confidence interval; NA, not available; OR, odds ratio.

**Table 4:** Association of genotypes and alleles with hospital mortality of children with community-acquired pneumonia

Serial no.	Genotype	Expired ( *n* = 22)	Survived ( *n* = 308)	Crude OR (95% CI)	*p* -Value
1	A1/A1 ( *n* = 152) Others	0 22	152 156	0.02 (0.001–0.37)	**< 0.001**
2	A2/A2 ( *n* = 61) Others	14 8	47 261	**9.7 (3.86–24.45)**	**< 0.001**
3	A3/A3 ( *n* = 11) Others	2 20	9 299	3.3 (0.67–16.42)	0.16
4	A4/A4 ( *n* = 7) Others	2 20	5 303	6.06 (1.10–33.22)	0.04
5	A1 ( *n* = 379) Others	1 21	226 82	0.01 (0.002–0.13)	**< 0.001**
6	A2 ( *n* = 177) Others	17 5	100 208	**7.07 (2.53–19.72)**	**< 0.001**
7	A3 ( *n* = 45) Others	5 17	29 279	2.8 (0.97–8.23)	0.10
8	A4 ( *n* = 59) Others	3 19	47 261	0.87 (0.24–3.08)	0.99

Abbreviations: CI, confidence interval; OR, odds ratio.

Routine laboratory findings were compared between survived and expired cases of CAP. Mean C-reactive protein (CRP) (mg/dL) levels were significantly higher 17.52 ± 9.79 ( *p* 0.004) in expired cases. Similarly, in A2/A2 genotype the CRP levels were 16.64 ± 5.30 ( *p* 0.01), significantly higher in expired cases. No significant difference was observed in hemoglobin, platelets, and total leukocyte cells among expired CAP cases.

Unconditional logistical regression analysis showed that A1/A2 and A1/A3 genotype was protective and A2/A2 genotype of *IL-1RA* gene was associated with increased risk for CAP children controlling for SES, PCV and measles vaccination, and height for age ≤ –3 SD *Z* score of WHO growth chart ( Table 5 ).

**Table 5:** Unconditional logistical regression model to assess association of genotypes of *interleukin-1 receptor antagonist* gene complications with community-acquired pneumonia children controlling for sociodemographics variables

Variables	Case/Control ^ref^		
	Coding	Adjusted OR (95% CI)	*p* -Value
A1/A2	1 = Present ( *n* = 35) 2 = Absent ( *n* = 295)	0.38 (0.22–19.72) reference	**< 0.001**
A1/A3	1 = Present ( *n* = 9) 2 = Absent ( *n* = 321)	0.19 (0.04–0.29) reference	**< 0.001**
A2/A2	1 = Present ( *n* = 61) 2 = Absent ( *n* = 269)	7.5 (2.9–19.7) reference	**< 0.001**
PCV vaccination	1 = yes ( *n* = 39) 2 = no ( *n* = 291)	0.09 (0.05–0.14) reference	**< 0.001**
Measles	1 = yes ( *n* = 84) 2 = no ( *n* = 246)	0.53 (0.33–0.85) reference	0.008
Socioeconomic status	Lower ( *n* = 169)	4.6 (2.8–7.5)	**< 0.001**
	Middle/Upper ( *n* = 161)	reference	
Height for age	≥ –2 SD ( *n* = 155)	reference	0.05
	–3 SD < WAZ ≤ –2 SD ( *n* = 66)	0.69 (0.38–1.26)	
	≤ –3 SD ( *n* = 109)	1.4 (0.90–2.39)	

Abbreviations: CI, confidence interval; OR, odds ratio; PCV, pneumococcal conjugate vaccine; SD, standard deviation; WAZ, weight-for-age.

## Discussion

The current case–control study was done in Northern India population with the objective to assess the association of *IL-1RA* gene polymorphism in children aged 2 to 59 months with CAP and the secondary objective was to assess the association of gene polymorphism with mortality among hospitalized cases. In our study, A2/A2 genotype and A2 allele of the *IL-1RA* gene were found to be associated with an increased risk of CAP children. In addition, it was also found that A1/A2 and A1/A3 genotype and A1 allele of *IL-1RA* gene polymorphism to have protective functions in CAP children. Cases with A2/A2 genotype had significant association with CAP mortality as compared with other genotypes.

In the current study, A2/A2 genotype and A2 allele of the *IL-1RA* gene were found to be associated with seven times increased risk of CAP children. Similar findings have been reported in various studies. 3 21 A study reported in a similar study setting that A2/A2 genotype or A2 allele of *IL-1RA* gene was associated with AOs in children with severe CAP. Similarly, another study had reported that A2/A2 genotype was associated with the risk of severe sepsis in the adult population. In our study, A1 allele was found to be protective for the CAP children. A similar finding was reported by Patwari et al that A1 allele was protective for the development of ARDS and the need for positive pressure ventilation in children with CAP. 2 In contrast to this study, a study reported A1 allele as a risk factor for the development of juvenile myositis in Caucasian populations. 22 The genotype A2/A2 and allele A2 of *IL-1RA* gene were significantly associated with mortality in CAP children. Similar finding was reported by Arlanich et al 21 in the adult population and Awasthi et al in pediatric North Indian population.

Because of their high polymorphic content, VNTRs constitute useful tools in population genetic studies in understanding population and ethnic variations. Allelic association studies are in the progress with several chronic inflammatory and degenerative diseases in *IL-1/IL-1RA* may be involved. In the long run, these studies may help in determining disease susceptibility and clinical management of patients. The IL-1 gene family includes IL-1α, IL-1β, and *IL-1RA* . The gene encoding these proteins is located on the short arm of chromosome 2. *IL-1RA* is a competitive inhibitor of IL-1 bioactivity.

### Prevention and Management of Pneumonia

Those children who have A2/A2 genotype are at higher risk of AO and hence may be referred to higher facility for management.

Cases with A2/A2 genotype also have increased levels of IL-1RA which possibly result in AO by stimulating the cytokines cascade. 3 Anakinra is a recombinant anti-inflammatory drug. Mechanism of anakinra against IL-1 is regulated by suppressing the natural binding of IL-1 to IL-1ra receptor and IL-1 accessory portion (IL-1Acp) that are present on the T cell membranes. 23 24 Therefore, the use of this drug reduces the inflammatory response of the disease. Studies have reported that the utilization of anakinra reduced the requirement of ventilation in coronavirus disease patient with pneumonia. 23 Therefore, further research can be done to see if those cases with A2/A2 genotype with AO can be benefitted by anakinra drug.

### Strength and Weakness

We have done the genotyping and allele distribution of VNTRs loci in *IL-1RA* gene in North Indian populations in cases and controls both so that our work is generalizable and has internal and external validity. Earlier study done in the same area did not recruit controls. We did not assess serum IL-1RA or IL-6 levels in cases and controls, which is a limitation of our study. Further genome-wide association studies and their functional implications with IL-1RA gene polymorphisms are required with different study designs in various populations.

## Conclusion

The A2/A2 genotypes and A2 and A4 allele of the *IL-1RA* gene were associated with an increased risk of CAP children. The A1/A2 and A1/A3 genotype and A1 allele were found to be protective for CAP children. A2/A2 genotype was significantly associated with CAP mortality. Further genome-wide association studies and their functional implication with *IL-1RA* gene polymorphism are required with different study designs in various populations.
